# Climate warming will test the limits of thermal plasticity in rainbow trout, a globally distributed fish

**DOI:** 10.1093/conphys/coaf034

**Published:** 2025-05-28

**Authors:** Nicholas Strowbridge, Matthew J H Gilbert, Yangfan Zhang, David C H Metzger, Jessica L McKenzie, Lais Lima, Anthony P Farrell, Nann A Fangue, Patricia M Schulte

**Affiliations:** Department of Zoology, University of British Columbia, 6270 University Blvd. Vancouver, BC V6T 1Z4, Canada; Department of Zoology, University of British Columbia, 6270 University Blvd. Vancouver, BC V6T 1Z4, Canada; Department of Zoology, University of British Columbia, 6270 University Blvd. Vancouver, BC V6T 1Z4, Canada; Faculty of Land and Food Systems, University of British Columbia, 2357 Main Mall Vancouver, BC V6T 1Z4, Canada; Department of Zoology, University of British Columbia, 6270 University Blvd. Vancouver, BC V6T 1Z4, Canada; Department of Zoology, University of British Columbia, 6270 University Blvd. Vancouver, BC V6T 1Z4, Canada; Department of Wildlife, Fish and Conservation Biology, University of California Davis, 455 Crocker Lane Davis, CA, USA, 95616; Department of Biology, San Diego State University, 5500 Campanile Dr San Diego, CA 92182, USA; Department of Zoology, University of British Columbia, 6270 University Blvd. Vancouver, BC V6T 1Z4, Canada; Faculty of Land and Food Systems, University of British Columbia, 2357 Main Mall Vancouver, BC V6T 1Z4, Canada; Department of Wildlife, Fish and Conservation Biology, University of California Davis, 455 Crocker Lane Davis, CA, USA, 95616; Department of Zoology, University of British Columbia, 6270 University Blvd. Vancouver, BC V6T 1Z4, Canada

**Keywords:** acclimation, aerobic scope, Ct_max_heart rate, hypoxia tolerance, MMR*, Oncorhynchus mykiss*rainbow trout, RNA-seq, thermal tolerance

## Abstract

Phenotypic plasticity is thought to be critical in allowing organisms to cope with environmental change, but the factors that limit this plasticity are poorly understood, which hampers predictions of species resilience to anthropogenic climate change. Here, we ask if limited plasticity in key traits constrains performance at high temperatures, using two California hatchery strains of rainbow trout (*Oncorhynchus mykiss*). Aerobic and anaerobic metabolic performance declined at a high but ecologically relevant acclimation temperature (24°C), suggesting performance cannot be maintained at this temperature, despite acclimation. Similarly, while both whole-organism thermal tolerance and hypoxia tolerance improved with acclimation to moderately elevated temperatures, compensation was limited at the highest acclimation temperature. These limits at the whole-organism level were aligned with limits at lower levels of biological organization. At the organ level, absolute scope to increase heart rate with acute warming (Δƒ_Hmax_) did not increase between the upper two acclimation temperatures, and the safety margin for cardiac performance decreased at the highest acclimation temperature. At the cellular level, at 24°C, there were transcriptomic changes in the heart consistent with a cellular stress response. These limits across multiple levels of biological organization were observed under conditions that are ecologically relevant at the southern end of the species range, which suggests that thermal plasticity is likely insufficient to buffer rainbow trout against even modest anthropogenic warming in these regions.

## Introduction

Increases in global temperatures are already negatively affecting biodiversity (IPCC, 2023), particularly in freshwater ecosystems, where rates of extinction are approximately five times higher than those in terrestrial or marine habitats (Jenkins, 2003; Comte and Olden, 2017a). In aquatic ecosystems, increases in temperature are also associated with increased frequency of hypoxia (Diaz and Breitburg, 2009; Earhart *et al.,* 2022). This combination of stressors is particularly challenging for fish because routine metabolic oxygen requirements increase exponentially with temperature while oxygen availability declines (Earhart *et al.,* 2022). Although some species can move to cooler and more normoxic habitats (Heino *et al.,* 2009; Chen *et al.,* 2011; Comte *et al.,* 2013), many organisms and particularly those in freshwater ecosystems, have limited opportunities for such movements due to habitat fragmentation and limited connectivity (Reid *et al.,* 2019). To survive *in situ*, these organisms must instead either recruit existing phenotypic plasticity or adapt via selection on standing genetic variation (Barrett and Schluter, 2008; Fuller *et al.,* 2010; Somero, 2010). However, with climate change proceeding too rapidly for many organisms to track environmental changes through adaptation (Radchuk *et al.,* 2019; Morgan *et al.,* 2020), more rapid plastic responses that occur over days and weeks may be crucial determinants of organismal resilience to anthropogenic environmental change (Williams *et al.,* 2008; Gunderson *et al.,* 2017; Fox *et al.,* 2019; Morley *et al.,* 2019).

Salmonid fishes have great ecological, economic and cultural importance, and many salmonid species are already experiencing declines as a result of factors including increased water temperature, hypoxic events and the increased frequency and intensity of heatwaves and drought (Busby *et al.,* 1996; Grant *et al.,* 2019). For example, the native range of rainbow trout in Western North America has already contracted in response to habitat loss and climate change (Moyle *et al.,* 2017). Thus, the future resilience of salmonids such as rainbow trout to anthropogenic warming likely depends both on the ability to maintain performance at elevated temperatures over the long term and on the ability to withstand acute exposure to brief periods of extremely high temperatures (*e.g.* heat waves). Consequently, there is a pressing need to understand the thermal limits and extent of plasticity in both medium-term performance metrics and short-term tolerance to warming.

At the organismal level, in fishes there is substantial evidence that long-term exposure to high temperatures can ultimately limit aerobic capacity and thus affect key fitness-related traits that depend on aerobic processes such as growth, swimming performance and reproduction (Claireaux and Lefrançois, 2007; Schulte, 2015; Jutfelt *et al.,* 2021). Indeed, the thermal optimum and pejus temperatures for aerobic scope are strongly correlated with environmental temperatures at the warm end of the species range across fishes (Payne *et al.,* 2016). This suggests that these traits might constrain species distribution limits, supporting the critical importance of aerobic scope for fitness, the evidence for which is particularly compelling for salmonid fishes (Eliason *et al.,* 2011). Less well-studied in the context of climate change resilience, but also likely to be an important determinant of fitness, is anaerobic performance (Sørensen *et al.,* 2014; Dressler *et al.,* 2023), because glycolytic metabolism is needed to support high-intensity, life-supporting activities such as avoiding predators, competing with conspecifics and navigating high flow areas such as rapids during migration. In addition to these performance traits, the capacity to tolerate short, intense adverse high temperature events may be critical for resilience to events such as heatwaves. Indeed, multiple studies have shown strong correlations between acute tolerance and thermal habitats, suggesting links between these traits and fitness in a changing environment (Sunday *et al.,* 2012, 2019).

The extent of plasticity in these critical whole-organism performance and tolerance traits may be an important determinant of climate-change resilience, but this beneficial plasticity has limits (Gunderson and Stillman, 2015). Thus, understanding the ceilings of plasticity across key traits is likely to be crucial for modelling and predicting how organisms will respond to anthropogenic environmental change (Gunderson and Stillman, 2015; Gunderson *et al.,* 2017; Morley *et al.,* 2019; Pottier *et al.,* 2021). Among many questions that remain about the factors that limit plasticity in performance and tolerance to high temperatures, the potential for cross-tolerance is a particularly poorly understood aspect of plasticity. Cross-tolerance is a phenomenon in which exposure to one stressor increases tolerance to an alternative stressor. For fishes, the potential for thermally mediated cross-tolerance to hypoxia may be of particular importance because of the direct physiological interactions between these two stressors (Earhart *et al.,* 2022). However, there is little available data with which to assess the extent of high-temperature induced tolerance to hypoxia in fishes or the limits to this plasticity (Collins *et al.,* 2021; Earhart *et al.,* 2022).

Temperature exerts its impacts at the biochemical reaction rate. Yet, the mechanisms via which the effects of temperature at the molecular level scale up to affect processes at higher levels of biological organization remain elusive (Pörtner, 2002; Schulte, 2015; Rezende and Bozinovic, 2019; Chung and Schulte, 2020). Several studies suggest that thermal limits are lower at higher levels of organization compared to limits at the cellular and subcellular levels (Pörtner, 2002; Rezende and Bozinovic, 2019; Bozinovic *et al.,* 2020). However, evidence exists for other patterns depending on the traits, taxa and life stages examined (Gangloff and Telemeco, 2018; Iverson *et al.,* 2020). In salmonids, strong evidence suggests that cardiac capacity may be an important determinant of aerobic scope and organismal performance (Farrell, 2009; Farrell *et al.,* 2009; Casselman *et al.,* 2012; Anttila *et al.,* 2013) and that cardiac traits also show substantial capacity for thermal acclimation (Keen *et al.,* 2017; Gilbert and Farrell, 2021; Gilbert *et al.,* 2022). At the cellular level, gene expression patterns can be sensitive indicators of thermal stress (Jeffries *et al.,* 2018), reaching various thresholds as temperature rises, including beneficial acclimation responses across intermediate temperatures, and then various levels of stress responses with the onset of cellular stress.

Here, we examine potential thermal limits and limitations in thermal plasticity for traits across multiple levels of biological organization in two hatchery strains of rainbow trout (*Oncorhynchus mykiss*) from California to ask:

1) Is there evidence of a limitation on aerobic and glycolytic metabolic performance with acclimation to high temperature?
2) Does plasticity in heat tolerance and associated cross-tolerance to hypoxia reach a limit with acclimation to high temperature?
3) Are limits to thermal plasticity aligned with changes in cardiac function and gene expression?

Assessing thermal limits and the limits of plasticity across multiple biological levels within a single study, from whole animal, through organ systems, to individual tissues and gene expression is essential to not only determine mechanisms that may underlie thermal limitations, but also to mechanistically predict the extent to which a species or population is likely to thrive or suffer as climate change progresses (Mykles *et al.,* 2010; Somero, 2010).

## Materials and Methods

### Experimental animals and acclimations

We utilized juvenile fish (4–5 months of age) from two hatchery strains of Rainbow trout from California: the Shasta stain—a hatchery strain originating from a cross between the Hot Creek strain and Meader’s Trout farm rainbow trout and the Coleman strain—a hatchery strain originating from the Central Valley in California (Busack and Gall, 1980). Shasta fish were obtained from the American River Hatchery (Gold River, CA) and Coleman fish from the Moccasin Creek Hatchery (Moccasin, CA). We selected these strains because they originate towards the southern limits of the species’ natural range, and thus are presumed to be warm-adapted (Myrick and Cech, 2000; Verhille *et al.,* 2016), and they are widely stocked across California. In addition, both were available at the same time of year, allowing experiments to be conducted simultaneously. However, slight differences in spawn timing, growth rate and hatchery practices meant that the Shasta and Coleman strains differed considerably in body mass (~ 50 vs ~ 5 g; see Table S1 for all sample sizes, body mass and length for each experiment).

Fish were spawned in their respective hatcheries in November 2017 and offspring were transported to the Center for Aquatic Biology and Aquaculture (CABA) at the University of California, Davis in February, 2018 for experimental thermal acclimation and testing. All experiments were completed by April 2018 and were performed under approved animal use and care protocols A16–0329 from the University of British Columbia and 21 834 from the University of California, Davis. Sex cannot not be visually determined at this life stage and was not considered in further analyses.

Water temperature at both hatcheries was 11 ± 1°C, and after transport to CABA, fish were held at this temperature for several weeks prior to experimental acclimation. Temperature was then increased by 1.5°C^.^day^−1^ from 11°C to the relevant acclimation temperature (12, 18 or 24°C). Fish weights and lengths are available in Supplementary Table S1. We selected these acclimation temperatures to represent current mean late winter/early spring (12°C), mean spring extreme/summer (18°C) and extreme summer (24°C) temperatures for central and southern California freshwater ecosystems (Verhille *et al.,* 2016; Atkinson, 2018; Dressler *et al.,* 2023). The highest acclimation temperature is very close to the maximum temperature at which growth can be maintained (25°C) in these, and most other, strains of rainbow trout (Myrick and Cech, 2000; Adams *et al.,* 2022). Fish were acclimated for at least three weeks prior to the beginning of the testing period and all testing was completed within 5 weeks of the beginning of the acclimation period.

### Whole-animal respirometry

Whole-animal metabolic rates were measured in fully acclimated fish, and thus we did not estimate the extent of plasticity in these traits, but rather sought to determine whether fish could maintain aerobic and anaerobic performance across the range of acclimation temperatures. An established and standardized testing protocol, the integrated respiratory assessment protocol (IRAP) (Zhang *et al.,* 2017), was used to measure standard metabolic rate (SMR) maximum metabolic rate (MMR) and absolute aerobic scope (AAS) as indicators of aerobic performance and excess post-exercise oxygen consumption (EPOC) as an indicator of anaerobic performance (Birnie-Gauvin *et al.,* 2023), as well as providing an indicator of hypoxia tolerance, the critical partial pressure of oxygen (P_crit_) (Claireaux *et al.,* 2013). P_crit_ is the minimum oxygen tension at which SMR can be maintained aerobically and is an indicator of whole-animal oxygen extraction capacity (Ultsch and Regan, 2019). Fish were housed in individual respirometry chambers (7 fish per trial), and an additional empty chamber measured the background *Ṁ*O_2_. The volume of the respirometer was matched to the fish body mass (water volume:fish mass *=* 50:1 (Coleman) & 27:1 (Shasta)) to ensure a high signal-to-noise ratio for PO_2_ measurements (Claireaux *et al.,* 2013). All eight respirometers were immersed in a ~ 300 L bath at the acclimation temperature and PO_2_ was maintained by continuously circulating the bath water through an aerated gas exchange column.

Fish were fasted for at least 48 h before testing to ensure a post-prandial state for measuring SMR. First, fish were chased to exhaustion (gauged by failure to respond to the stimulus) individually by hand in a 20-L bucket before transfer to the respirometer with a standardized 1-min air exposure. This protocol was used to elicit MMR because it can be performed more rapidly than aerobic swimming to exhaustion, although it may result in a slightly lower estimate of MMR than U_crit_ methods in salmonids (Hvas and Oppedal, 2019; Raby *et al.,* 2020). However, in our hands the two methods provide similar results (Zhang *et al.,* 2020) and in the current study we achieved very similar MMR to those obtained by U_crit_ testing in other studies, (Hvas and Oppedal, 2019; Raby *et al.,* 2020) suggesting that any under-estimate was minor. In addition, the use of a chase protocol is likely to maximally recruit anaerobic metabolism, lending support to the use of EPOC as an indirect indicator of anaerobic capacity or performance. The respirometers received an intermittent water flow of aerated water from the bath water that consisted of open flush, closed stabilization and closed measurement periods. During the latter, the fish depleted the oxygen within their respirometer and the decline in PO_2_ was used to calculate *Ṁ*O_2_. Intermittent flushing ensured water PO_2_ in the respirometers was maintained above 70% air saturation during MMR and SMR measurements.

Across the acclimation temperatures, the flush cycle used during recovery immediately after exhaustion was a 20-s flush, a 40-s stabilization and a 120-s measurement. This cycle captured the maximum *Ṁ*O_2_ (*Ṁ*O_2max_). As *Ṁ*O_2_ decreased with recovery, the cycle was changed to either a 75-s flush, a 120-s stabilization and a 780-s measurement for the Coleman strain or 90-s flush, a 95-s stabilization and a 360-s measurement for the Shasta strain. The different measurement cycles were to optimize the signal-to-noise ratio, and were selected based on results from prior work in this species (Zhang *et al.,* 2018b).

After two days of monitoring, which is required to obtain reliable estimates of SMR (Chabot *et al.,* 2016), the IRAP ended with a hypoxia challenge test that measured P_crit_ by making the respirometers progressively hypoxic by gradually reducing the level of oxygen in the respirometers. PO_2_ in the respirometer bath was reduced in a controlled fashion by bubbling nitrogen through the gas exchange column such that over a 45-min period each flush cycle progressively reduced the respirometer PO_2_ to a moderate level of hypoxia for rainbow trout (i.e. about twice the expected P_crit_). The deoxygenation rate was then slowed (to 0.15 ± 0.02% air saturation min^−1^) until the fish lost equilibrium, after which the protocol was immediately terminated by removing fish from the respirometer chamber.

*Ṁ*O_2_ was continuously and automatically calculated using the rate of decline in the respirometer PO_2_ during the closed phase using signals from individual optical oxygen probes (at a 1-Hz sampling rate; Robust Oxygen Probe OXROB3, Pyroscience, Germany & Oxygen Minisensor, PreSens, Germany) equipped to each respirometer chamber. Optodes were calibrated to 0% saturation (water saturated with sodium sulphite and bubbled with nitrogen gas) and 100% saturation (fully aerated water) before each trial.

*Ṁ*O_2max_ (MMR) was assigned as the peak *Ṁ*O_2_ measurement post-exhaustion, which was typically seen during the first 120-s measurement cycle. The slope of the decrease in PO_2_ over time met a minimum requirement for linearity (i.e. *R*^2^ > 0.95) to calculate *Ṁ*O_2_. A sequential interval regression algorithm identified the highest *Ṁ*O_2_ value using Eqn 1.


(1) \begin{equation*} \dot{\mathrm{M}}{\mathrm{O}}_2=\left[\frac{{d_{DO}}_{\left[i,\left(i+a\right)\right]}}{{d_t}_{\left[i,\left(i+a\right)\right]}}\ast \left({V}_r-{V}_f\right)\ast{S}_O\right]/\left(t\ast{M}_f\right) \end{equation*}

where the unit for *Ṁ*O_2_ is mg O_2_ h^−1^ kg^−1^, ${{d}_{DO}}\!\left/ \!{{d}_t}\right.$ is the change in O_2_ saturation over time, *V_r_* is the respirometer volume, *V_f_* is the fish volume (estimated from body mass assuming neutral density; 1 g = 1 mL), *S_o_* is the solubility of O_2_ in freshwater at 1 atm, *t* is a time constant of 3600 s h^−1^, *M_f_* is fish mass, *a* is the sampling window duration (s) and *i* is one PO_2_ sample forward from the end of previous sampling window at a sampling frequency of 1 Hz.

SMR was assigned as the 20% quantile (q0.2) of *Ṁ*O_2_ values recorded over two diurnal cycles (excluding the first 12-h of measurements as a recovery period (Chabot *et al.,* 2016)). Typically, over 200 individual *Ṁ*O_2_ values per fish were used for the SMR estimate, which provided a reliable baseline for calculating other IRAP indices (i.e. AAS, total EPOC and P_crit_). AAS was calculated as the difference between *Ṁ*O_2max_ and SMR (AAS = *Ṁ*O_2max_—SMR (Claireaux *et al.,* 2005)). Excess post-exercise oxygen consumption (EPOC) after exhaustion was calculated from the sum of all *Ṁ*O_2_ values minus SMR until *Ṁ*O_2_ first returned to SMR plus 10% (Zhang *et al.,* 2018a). P_crit_ was calculated from the *Ṁ*O_2_ values obtained during progressive hypoxia according to best practice recommendations (Claireaux and Chabot, 2016). The slower phase of progressive deoxygenation generated at least 10 *Ṁ*O_2_ data points before *Ṁ*O_2_ fell below SMR (Claireaux *et al.,* 2013).

All metabolic performance metrics (SMR, MMR, AAS, P_crit_ and EPOC) were analysed by ANCOVA with log_10_ transformed body mass as a covariate. As expected, respiratory metrics (SMR, *Ṁ*O_2max_, AAS, EPOC) varied with body mass and were thus presented as mass-centred least-squares means, which were analysed using Holm-Šidák post hoc comparisons between strains and acclimation temperatures (lsmeans package; (Lenth, 2016)). P_crit_ did not vary with body mass and was analysed by ANOVA with Holm-Šidák post hoc comparisons. All *Ṁ*O_2_ measurements were corrected prior to analysis by subtracting the corresponding background *Ṁ*O_2_ value (<5%). Background respiration was minimized by thoroughly disinfecting the entire apparatus with diluted household bleach for 30 min between IRAP trials.

### Acute heat tolerance test (critical thermal maximum)

Critical thermal maximum (CT_max_), which is the temperature at which a fish is no longer able to maintain equilibrium in response to rapidly increasing temperature, was used an index of acute thermal tolerance. CT_max_ Although the direct ecological relevance of CT_max_ remains uncertain (Desforges *et al.,* 2023), this measure is correlated with biogeographic range boundaries and with tolerance to more ecologically relevant rates of warming (Sunday *et al.,* 2012; Dahlke *et al.,* 2020).

Fish were fasted for 48 h before transfer to an experimental tank, where they were left undisturbed for 30 min before CT_max_ testing. All CT_max_ trials began at the intermediate acclimation temperature (18°C) and were conducted according to previously published methods (Beitinger *et al.,* 2000), except that ramping rate was 0.1°C min^−1^ to improve our ability to detect small differences in CT_max_ among groups (Strowbridge *et al.,* 2021) relative to the standardized rate of 0.3°C min^−1^. Note that prior studies have suggested minimal impacts on absolute CT_max_ across these ramping rates (Becker and Genoway, 1979). An aeration bar and circulation pumps were placed in the experimental tank to maintain >75% air saturation and to prevent thermal stratification of the water. During the trial, water temperature was progressively increased by 0.1°C min^−1^ until the fish was unable to maintain dorsoventral orientation (i.e. loss of equilibrium, LOE), which represents an inability to escape a life-threatening circumstance. CT_max_ was assigned as the temperature (°C) at LOE, at which point the fish was removed from the experimental tank, euthanized and measured for length and weight.

### Hypoxia challenge test (LOE_hyp_)

**Figure 1:**
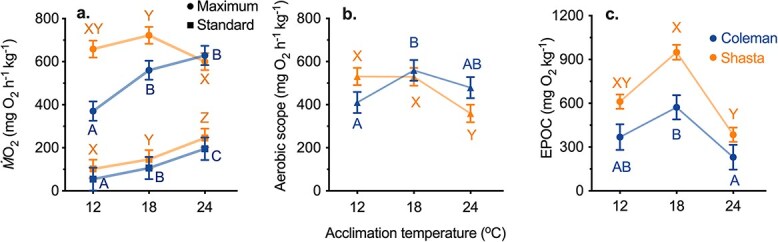
Whole-animal metabolic performance indices for two strains of Californian rainbow trout: Coleman (blue) and Shasta (orange) tested at their acclimation temperatures (12, 18 and 24°C). Oxygen uptake (*Ṁ*O_2_) rates were used to estimate maximum (**a**. circles MMR) and standard metabolic rate (a. squares SMR), absolute aerobic scope (**b**. AAS) and excess post-exercise oxygen consumption (**c**. EPOC). Dissimilar letters denote statistical differences (Coleman: A,B,C; Shasta: X,Y,Z) between temperatures within each strain and metric. Data were analysed by ANOVA with Holm-Sidak corrected post hoc comparisons (α = 0.05). Data presentation and post hoc analysis for *Ṁ*O_2_ data (a-c) were done using least square means (±SEM) at a common body mass to account for allometric scaling of metabolism.

To assess the effects of thermal acclimation on hypoxia tolerance, we measured the oxygen partial pressure (kPa) at which fish are unable to maintain equilibrium (LOE_hyp_) during an exposure to a progressive decrease in oxygen. LOE_hyp_ is a lethal oxygen level, reflecting a combination of limited oxygen supply capacity and the capacity for anaerobic metabolism (Nilsson and Östlund-Nilsson, 2008). Because test temperature can affect hypoxia tolerance in fish (Collins *et al.,* 2021; Earhart *et al.,* 2022), all trout were tested at their acclimation temperature (“Acclimated” in Fig. 1) and fish acclimated to 12°C were warmed acutely to either 18°C over 1 h or 24°C over 2 h and then tested (“Acute” in Fig. S1). We then compared the hypoxia tolerance between acutely exposed and acclimated fish to detect effects of warm acclimation. Water temperature for the hypoxia challenge test was maintained at the relevant temperature ± 1°C throughout the trial.

Prior to each LOE_hyp_ trial, fish were fasted for 48 h before transfer to an experimental tank, where they were left undisturbed for 30 min before testing. During the trial dissolved oxygen (DO, % air saturation) was progressively decreased at a rate of ~1.5% air saturation min^−1^ from ~90% to ~20% air saturation by bubbling nitrogen into the experimental tank. Small circulation pumps in the experimental tank prevented water stratification during the imposed hypoxia. At ~ 20% air saturation the ramping rate was reduced to 0.1% air saturation min^−1^ until the fish experienced loss of equilibrium. At this point, the fish was removed from the experimental tank, euthanized and measured for length and weight. Note that LOE_hyp_ represents an inverse measure of acute hypoxia tolerance; i.e. a lower LOE_hyp_ indicates higher hypoxia tolerance.

Differences in CT_max_ across acclimation temperature and strain were assessed by two-way ANOVA and Holm-Šidák adjusted pairwise comparisons. Differences in LOE_hyp_ across test temperatures, between acutely warmed and acclimated LOE_hyp_ within a strain, and across strains in both acutely warmed and acclimated fish, were analysed by three-way ANOVA and Holm-Šidák adjusted pairwise comparisons. LOE_hyp_ and CT_max_ data were transformed following the BoxCox procedure (MASS package) to meet assumptions of normality and homoscedasticity.

### Acute cardiac heat tolerance

Acute cardiac heat tolerance was assessed by measuring maximum heart rate (ƒ_Hmax_) during acute warming (Casselman *et al.,* 2012; Gilbert *et al.,* 2024). Anaesthetized fish (stage III anaesthesia; tricaine methanesulfonate; 150 mg/L MS-222 buffered 1:1.5 with NaHCO_3_) were placed in a foam trough and two cutaneous electrocardiogram (ECG) recording electrodes were placed over the heart, one near the left pectoral fin and one slightly more anterior on the right ventral side of the body. The gills were continuously irrigated with a maintenance dose of buffered MS-222 (60 mg L^−1^) throughout the warming trial. Fish were given intra-peritoneal injections (to a total volume of 3 ml kg^−1^ in 0.8% NaCl solution) of atropine (1.2 mg kg^−1^; to remove cholinergic inhibition of the heartbeat) and isoproterenol (4 ug kg^−1^; to stimulate cardiac β-adrenoreceptors) to achieve ƒ_Hmax_. The warming protocol began 15 min after these injections when the heartbeat had stabilized at the ƒ_Hmax_. Warming started at 12°C and occurred in increments of 1°C every 6 min (10°C h^−1^), continuing until the heartbeat became arrhythmic, as previously described (Chen *et al.,* 2015). Fish then were euthanized with an overdose of MS-222 followed by pithing.

Automated heartbeat detection (Labchart v.8, ADInstruments) was used to calculate ƒ_Hmax_ from a continuous recording of the ECG, using the final 1-min period of each 6-min temperature increment. The peak ƒ_Hmax_ reached during acute warming, the temperature at peak ƒ_Hmax_ (T_peak_) and the temperature at the first cardiac arrhythmia (T_arr_) were assessed as previously described (Chen *et al.,* 2015). The ability to increase ƒ_Hmax_ during acute warming (Δƒ_Hmax_) was calculated from the difference between ƒ_Hmax_ at the initial test temperature (12°C) and peak ƒ_Hmax_. Differences in Δƒ_Hmax_, peak ƒ_Hmax_, T_peak_ and T_arr_ among acclimation temperatures within a strain were assessed by ANOVA and Holm-Šidák adjusted pairwise comparisons. Differences in ƒ_Hmax_ during warming from 12 to 20°C between acclimation temperatures for each strain were assessed using linear mixed effect modelling (LMM; lme 4 package; Bates *et al.,* 2015), with fish ID included as a random factor, followed by Holm-Šidák adjusted post hoc comparisons of least-squared means between acclimation temperatures (lsmeans package; (Lenth, 2016)).

### Cardiac transcriptomics

Whole-transcriptome effects were examined in ventricular tissues of the Shasta and Coleman strains sampled after three weeks of acclimation at 12, 18 and 24°C. Six individuals from each strain and each acclimation temperature that had not been exposed to any tolerance assessment or other measurement were euthanized and the ventricle was dissected and stored in RNA*later*® (Sigma-Aldrich, St. Louis, Missouri). RNA was isolated using TRIzol® Reagent (Invitrogen, Carlsbad, California) followed by DNase digestion using the RNeasy Mini Kit on column protocol (QIAGEN, Hilden, Germany). DNase treated total RNA was then sent to the Genome Québec Innovation Centre (Montréal, Québec) for library preparation and sequencing. Sequencing depth and library quality information is available in Supplementary Table S2. The resulting raw reads were assessed for quality using FastQC (Andrews, 2010). rRNAs were removed using SortMeRNA (Kopylova *et al.,* 2012), and quality-base trimming and adapter removal was performed with Trimmomatic (Bolger *et al.,* 2014). Reads were then aligned to a pre-assembled *rainbow trout* transcriptome (assembly Omyk_1.0; https://www.ncbi.nlm.nih. gov/genome/196) using Bowtie2 (Langmead and Salzberg, 2012) and raw read counts per transcript per sample were generated using RSEM (Li and Dewey, 2011). Read counts were then filtered to exclude any transcript for which an individual(s) showed expression counts less than 10. The quality of the data was further assessed by testing for statistical outliers using the robust principal component analysis (PCA) method (Chen *et al.,* 2020). From this analysis, the sample X12C_CH5 from the Coleman 12°C was identified as an outlier and was removed from further analyses. Subsequent PCA for all genes was conducted using the prcomp() R function. Tests of differential expression were conducted using edgeR v3.40.2 (Robinson *et al.,* 2009; McCarthy *et al.,* 2012). All code is provided in the Supplementary data repository (https://doi.org/10.5683/SP3/O0JVPC).

Genes with a *q*-value <0.05 were considered differentially expressed between groups. Soft clustering of gene expression patterns was performed using Mfuzz v2.58 (Kumar and Futschik, 2007). This method groups genes with similar expression profiles and assigns a membership value to each gene within a cluster. Soft clustering was conducted separately for each population separately. The optimal number of clusters was determined to be 7 clusters for Coleman and 8 clusters for Shasta. Only genes with a minimum membership value of 0.7 were used for further analysis. Gene ontology category (GO) annotations for orthologous human genes were used for GO enrichment analysis. Human orthologs for rainbow trout genes were identified using BLAST with an e-value threshold of 1e-7. Human GO annotations were downloaded from the Ensemble Biomart. GO enrichment analysis was performed using goseq v1.5 (Young *et al.,* 2010) by comparing the GO annotation list of differentially expressed genes for a given comparison to a background list containing GO annotations for all genes used in the differential expression analysis and also accounting for gene length bias. All raw sequencing data are available in NCBI under BioProject ID: PRJNA759286.

## Results

### Aerobic scope and anaerobic performance decline at high temperature

Consistent with previous work in salmonids (Farrell, 2016), standard metabolic rate (SMR) increased with acclimation temperature in both strains (Fig. 1a ANCOVA, *P* ≤ 0.005), whereas maximum aerobic metabolic rate (MMR, as estimated by maximum oxygen uptake; *Ṁ*O_2max_) showed evidence of either reaching a plateau or declining at higher temperatures (See Supplementary file DataS1 at https://doi.org/10.5683/SP3/O0JVPC for all metabolic rate data). Specifically, MMR in the Coleman strain increased between the 12 and 18°C acclimation temperatures (Fig. 1a ANCOVA Coleman_12v18_: *P* < 0.001), but plateaued at 24°C, (Coleman_18v24_: *P* = 0.427). MMR in the Shasta strain, in contrast, did not increase significantly from 12 and 18°C (Shasta_12v18_: *P* = 0.611) and then decreased significantly at 24°C (Shasta_18v24_: *P* = 0.029). Similarly, AAS (*Ṁ*O_2max_—SMR; Fig. 1b) was also significantly lower at the highest acclimation temperature (24°C) for the Shasta strain (Shasta_18v24_: *P* < 0.001) and plateaued or declined slightly in the Coleman strain (Coleman_18v24_: *P* = 0.702) (Fig. 1b). Together, these data also suggest that the aerobic metabolic performance of fish from the Shasta strain is somewhat more sensitive to high temperature compared to the Coleman fish. These differences could be due to effects of early rearing environment in their respective hatcheries, the 10-fold difference in body mass between the strains (Supplementary Table S1), genetic differences between the strains or some combination of these factors.

**Figure 2:**
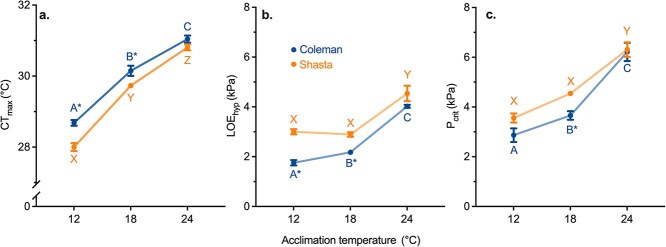
Upper limits for thermal plasticity in whole-animal heat and hypoxia tolerance for two Californian strains of rainbow trout: Coleman (blue) and Shasta (orange). Data are shown for acute heat tolerance (CT_max_; critical thermal maximum; **a**.), oxygen partial pressure at loss of equilibrium (LOE_hyp;_**b**.) and the critical oxygen partial pressure for standard metabolic rate (P_crit_; **c**.). For LOE_hyp_ and P_crit_ a lower value indicates better hypoxia tolerance. LOE_hyp_ and P_crit_ were assessed in fish at their acclimation temperature. Dissimilar letters indicate significant differences within a strain between acclimation temperatures (Coleman: A,B,C; Shasta: X,Y,Z). Asterisks (*) indicate significant differences between strains. Data were analysed by ANOVA with Holm-Sidak corrected pairwise comparisons (α = 0.05). Data are presented as mean ± SEM. See supplemental Fig. S1 for LOE_hyp_ data for fish acclimated to 12°C and tested at 18 and 24°C, which allows assessment of the effects of acclimation on this trait.

Measures of oxygen consumption during the recovery period following a bout of exhaustive exercise (i.e. EPOC) indirectly estimate the use of glycolytic metabolism during the exercise bout (Gaesser and Brooks, 1984). Our data supported the prediction that EPOC, like AAS, has a bell-shaped curve with temperature, with EPOC declining above an optimum temperature. Specifically, in both strains the tendency was for EPOC to be higher at 18°C than at 12°C (although this difference did not reach significance in post hoc tests; Fig. 2d; Coleman_12v18_: *P* = 0.163, Shasta_12v18_: *P* = 0.204), and then EPOC decreased appreciably at 24°C in both strains (Coleman_18v24_: *P* < 0.001, Shasta_18v24_: *P* < 0.001). This result suggests anaerobic performance was limited or that mechanisms of post-exercise recovery are significantly altered at the highest acclimation temperature in both strains.

### Plasticity in CTmax declines with high-temperature acclimation

Consistent with the expectation that CT_max_ is quite plastic with thermal acclimation in fishes (Comte and Olden, 2017b; Morley *et al.,* 2019; Adams *et al.,* 2022), CT_max_ increased with acclimation temperature in both strains of rainbow trout (Fig. 2a, two-way ANOVA temperature: *P* = 2.00 × 10^−16^, Temperature×Strain: *P* = 2.55 × 10^−1^; online file DataS1 at https://doi.org/10.5683/SP3/O0JVPC). The extent of plasticity in CT_max_ is typically quantified using the Acclimation Response Ratio, (ARR; ΔCT_max_/Δaccl. temp.), with an ARR = 1 suggesting complete thermal compensation. However, ARR was much lower than 1 across all acclimation temperatures and declined with increasing acclimation temperature (Coleman: ARR_12v18_ = 0.24, ARR_18v24_ = 0.15; Shasta: ARR_12v18_ = 0.29, ARR_18v24_ = 0.18). Thus, the thermal safety margin (TSM; the difference between CT_max_ and the acclimation temperature) more than halved across increasing acclimation temperatures (TSM Coleman 16.7 ± 0.1, 12.1 ± 0.1 and 7.0 ± 0.1°C; TSM Shasta: 16.0 ± 0.1, 11.7 ± 0.5, 6.8 ± 0.1°C (mean ± SEM) for the 12, 18 and 24°C acclimation temperatures, respectively; two-way ANOVA strain *P* < 0.0001, acclimation temperature *P* < 0.0001, interaction *P* = 0.0812).

Despite the two strains having similar limits to plasticity in CT_max_, their absolute CT_max_ differed significantly (Fig. 1a, two-way ANOVA Strain: p = 9.49 × 10^−7^) with the Coleman strain having a higher CT_max_ at the lower and intermediate acclimation temperatures (Strain_12_: *P* = 1.86 × 10^−4^; Strain_18_: *P* = 4.60 × 10^−3^), but not at the highest acclimation temperature (Strain_24_: *P* = 5.92 × 10^−2^). This superior performance of the Coleman fish at the lower acclimation temperatures could reflect a genetic difference in tolerance between the strains, impacts of differences in early rearing environment or the 10-fold difference in body mass between the strains (Supplementary Table S1) or some combination of these factors. However, with meta analyses providing limited evidence for developmental plasticity in CT_max_ (Pottier *et al.,* 2022) and CT_max_ not being strongly influenced by body size in most fish species, including rainbow trout (Fangue *et al.,* 2006; O’Donnell *et al.,* 2020; Strowbridge *et al.,* 2021), a potential influence of genetic differences between the strains is plausible.

### Partial compensation of hypoxia tolerance with thermal acclimation

LOE_hyp_ was significantly affected by acclimation temperature and differed between strains (Fig. 2b; Temperature: *P* < 0.001, Strain: *P* < 0.001, Interaction: *P* < 0.001). Between acclimation temperatures of 12 and 18°C, LOE_hyp_ either did not change (Shasta strain; *P* = 0.543) or increased slightly (by 24%; Coleman strain; *P* < 0.001), suggesting complete or nearly complete compensation. In contrast, LOE_hyp_ increased substantially between 18 and 24°C (by 85% in the Coleman strain; *P* < 0.001 and 60% in the Shasta strain; *P* < 0.001), indicative of reduced tolerance. P_crit_ displayed similar patterns (Fig. 2c; ANOVA Temperature *P* < 0.001, Strain *P* = 0.003). Specifically, *P*_crit_ increased modestly (by ~ 30%) from 12–18°C (Coleman_12v18_: *P* = 0.047, Shasta_12v18_: *P* = 0.015, and by ~ 70% and 40% from 18–24°C in the Coleman and Shasta strains, respectively (Coleman_18v24_: *P* < 0.001, Shasta_18v24_: *P* < 0.001).

Estimating the extent to which thermal acclimation improves hypoxia tolerance (i.e. the extent of cross-tolerance) requires knowledge of the effects of acute exposure to high temperature because high temperature is expected to directly reduce hypoxia tolerance (Collins *et al.,* 2021; Earhart *et al.,* 2022). Thus, we additionally measured LOE_hyp_ for fish acclimated to 12°C that were then acutely exposed to either 18 or 24°C. As expected, these fish greatly reduced hypoxia tolerance when acutely exposed to a higher temperature (much greater LOE_hyp_; Fig. S1). To estimate the extent to which thermal acclimation improved hypoxia tolerance, we computed the improvement in LOE_hyp_ due to acclimation by subtracting the individual LOE_hyp_ of fish tested at their acclimation temperature from the mean of those acutely tested at each temperature. After thermal acclimation, LOE_hyp_ improved by ~30% regardless of acclimation temperature (Fig. S1). This degree of plasticity with thermal acclimation was, however, only sufficient to offset the acute effects of temperature on hypoxia tolerance at 18°C and not at 24°C (Fig. 2b). Together, the LOE_hyp_ and P_crit_ data clearly indicate that these two strains of rainbow trout were reaching a limit to their ability to maintain hypoxia tolerance at an acclimation temperature of 24°C.

Coleman trout had a lower LOE_hyp_ than Shasta trout at 12°C (LOE_hyp_: 42%, *P* < 0.001; *P*_crit_: 19%, *P* = 0.078) and lower LOE_hyp_ and *P*_crit_ at 18°C (LOE_hyp:_ 25%, *P* < 0.001; *P*_crit:_ 19%, *P* = 0.026). Thus, the Coleman strain had greater hypoxia tolerance over low and intermediate acclimation temperatures. Neither metric, however, differed between strains at the high acclimation temperature (LOE_hyp_*P* = 0.201; *P*_crit_*P* = 0.788). Strain comparisons must be made with caution because of differences in body size (Supplementary Table S1). Nevertheless, our observations are inconsistent with previous research on other fish species that indicates that larger individuals may be more hypoxia tolerant than smaller ones (Nilsson and Östlund-Nilsson, 2008; Roze *et al.,* 2013; Hines *et al.,* 2019) given a greater hypoxia tolerance was exhibited for the smaller Coleman strain. This observation suggests the potential for genetically or epigenetically mediated differences in hypoxia tolerance between the strains.

### Incomplete compensation of cardiac heat tolerance with thermal acclimation

**Figure 3:**
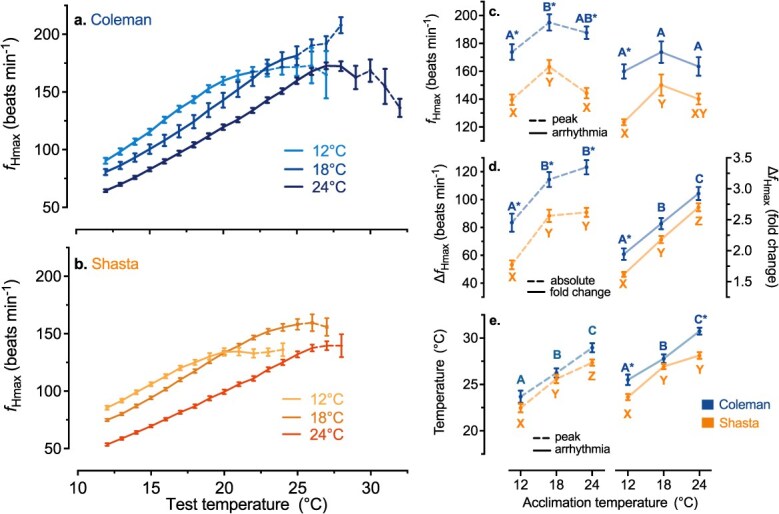
Acute cardiac heat tolerance as measured by the response of maximum heart rate (ƒ_Hmax_) during acute warming for two strains of Californian rainbow trout: Coleman (blue) and Shasta (orange) after acclimation to three ecologically relevant temperatures (12, 18 and 24°C). Acute warming of the Coleman (**a**.) Shasta (**b**.) strains increased ƒ_Hmax_ to a maximum (peak ƒ_Hmax_). Warm acclimation progressively reset ƒ_Hmax_ to a lower rate over most (12–20°C) of the acute temperature range (*n* = 8–11 per treatment group; Table S1). Broken lines in ƒ_Hmax_ curves indicate that individual fish had been removed from the average because their heartbeat became arrhythmic at a lower temperature. The peak ƒ_Hmax_ and ƒ_Hmax_ at arrhythmia (**c**.), the total absolute and relative (fold) increase in ƒ_Hmax_ (Δƒ_Hmax_; **d**.) and the temperature at peak ƒ_Hmax_ and the onset of arrhythmia (**e**.) are shown with significant differences between acclimation temperatures within a strain indicated by dissimilar letters (Coleman: A,B,C; Shasta: X,Y,Z). An asterisk (*) indicates a significant difference between strains within an acclimation temperature. The resetting of ƒ_Hmax_ between acclimation temperatures within a strain was assessed using linear mixed effect modelling and all other metrics were analysed by ANOVA with Holm-Sidak corrected post-hoc comparisons (α = 0.05). Data are presented as mean ± SEM.

Warm acclimation substantially lowered the maximum heart rate (ƒ_Hmax_) of fish acutely exposed to temperatures below 20°C, consistent with a compensatory resetting of pacemaker rates (Fig. 3a and b; LMM *P* < 0.001 for both strains), as seen in some other fish species including other strains of rainbow trout (Adams *et al.,* 2022; Gilbert *et al.,* 2022). Furthermore, acclimation from 12 to 18°C significantly increased the peak ƒ_Hmax_ attained with acute warming in both strains (Coleman_12v18_: *P* = 0.006, Shasta_12v18_: *P* = 0.008), again as seen in some other fish species and in other strains of rainbow trout (Gilbert and Farrell, 2021; Gilbert *et al.,* 2022). Yet, peak ƒ_Hmax_ either decreased (Shasta_18v24_: *P* = 0.029) or was unchanged (Fig. 3c; Coleman_18v24:_*P* = 0.303) between the 18 and 24°C acclimation groups, suggesting that by 24°C this form of cardiac plasticity had reach a limit.

The compensatory resetting of ƒ_Hmax_ and increase in peak ƒ_Hmax_ in both strains with acclimation temperature up to 18°C (*P* < 0.001) increased the absolute scope to increase heart rate with acute warming (Δƒ_Hmax_). However, Δƒ_Hmax_ did not increase further with acclimation to 24°C (Fig. 3d; Coleman_18v24:_*P* = 0.229, Shasta_18v24_: *P* = 0.749). Nevertheless, the relative ability to increase ƒ_Hmax_ (fold Δƒ_Hmax_) improved across all acclimation temperatures in both strains (Fig. 3d; *P* < 0.001).

Warm acclimation also increased the temperatures at which cardiac function showed signs of deterioration with acute warming. Specifically, the temperatures at which peak heart rate (T_peak_) and cardiac arrhythmia (T_arr_) occurred both increased substantially (~5°C) with acclimation temperature in both strains (Fig. 3e, ANOVA T_arr_: *P* < 0.001, T_peak_: *P* ≤ 0.027). However, the increase in T_arr_ between 18 and 24°C in the Shasta strain did not reach statistical significance in post hoc tests (Shasta T_arr18v24_: *P* = 0.080).

As expected, cardiac heat tolerance (as indicated by T_peak_ and T_arr_) occurred at temperatures below CT_max_ and like CT_max_ the ARR for T_arr_ was much lower than 1, indicating incomplete compensation (Coleman: ARR_12v18_ = 0.38, ARR_18v24_ = 0.50; Shasta: ARR_12v18_ = 0.55, ARR_18v24_ = 0.20). Consequently, the thermal safety margins for cardiac function were also lower than for CT_max_ and declined with increasing acclimation temperature (Coleman: TSM_12_ = 13.5 ± 0.6°C, TSM_18_ = 9.8 ± 0.5°C, TSM_24_ = 6.8 ± 0.4°C; Shasta: TSM_12_ = 11.6 ± 0.3°C, TSM_18_ = 8.9° ± 0.3C, TSM_24_ = 4.1 ± 0.4°C; mean ± SEM; two-way ANOVA: Temperature *P* < 0.0001; strain *P* < 0.0001; interaction *P* = 0.15).

### A cellular stress response is induced with high-temperature acclimation

**Figure 4:**
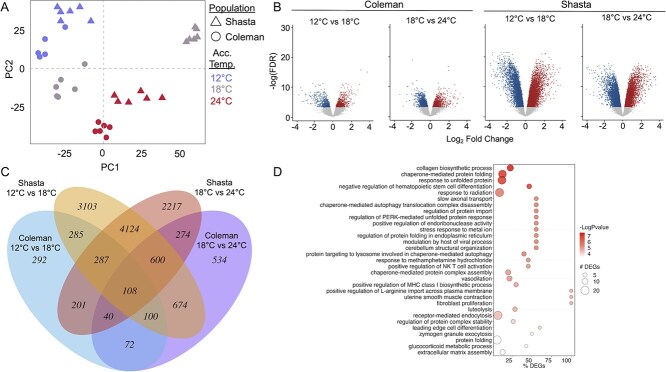
Gene expression in the cardiac ventricle of two strains of California Rainbow trout after acclimation to three ecologically relevant temperatures (12, 18 and 24°C). (**a**.) PCA of all expressed genes. (**b**.) Volcano plots displaying DEGs within each population, comparing the low and high acclimation temperatures to fish acclimated to 18°C. (**c**.) Shared and unique DEGs among populations and temperatures. (**d**.) GO analysis for DEGs between 18 and 24°C that are detected in both strains.

Whole-transcriptome analysis on cardiac ventricular tissue of thermally acclimated trout sampled at rest under normoxic conditions was used to assess whether acclimation at a high, but non-lethal, temperature results in a cellular stress response (see online DataS2 at https://doi.org/10.5683/SP3/O0JVPC for gene counts). PCA of all expressed genes (Fig. 4a) demonstrated that gene expression was highly distinct across acclimation temperatures. PC1, which accounted for 28% of the variation, was largely driven by differences between the strains (particularly at 18°C), along with a modest contribution of acclimation temperature, whereas PC2, which accounted for 18% of the variation, was largely associated with differences as a result of acclimation temperature (see Supplementary Fig. S2 for remaining principal components). At both high and low acclimation temperatures, the two strains had fairly similar gene expression patterns, but these diverged in the 18°C acclimated fish (Fig. 4a). Gene Ontology enrichment (GO) analysis of gene expression differences between strains at 18°C indicated enrichment of GO terms involved in ubiquitination (GO:0016567) and immune system processes (GO:0002376; GO: 0002250) for genes which had higher expression in the Shasta strain than in the Coleman strain (Supplementary DataS3 at https://doi.org/10.5683/SP3/O0JVPC). This suggests that the Shasta strain may be experiencing higher levels of cellular stress in the cardiac ventricle at 18°C, which is consistent with the lower tolerance and aerobic performance of the Shasta strain at 18°C (Figs 1 and 2). However, there was also significant enrichment of GO terms associated with protein folding (GO:0042026; GO:0060904) among genes that were expressed at lower levels in the Shasta strain, which highlights the complexity of these strain differences.

Across all temperatures, thermal acclimation had profound effects on the cardiac transcriptome of both strains, although greater differential expression was detected in the Shasta strain (Fig. 4b). Comparing the 12 and 18°C acclimation temperatures, there were 9281 and 1385 differentially expressed genes (DEGs) in the Shasta and Coleman strains, respectively (Fig. 4b and c). Similarly, between 18 and 24°C, there were 7851 DEGs in the Shasta strain and 2402 in the Coleman strain (Fig. 4b and c; Supplementary DataS4 at https://doi.org/10.5683/SP3/O0JVPC). Sequencing coverage was not statistically different between strains or among acclimation temperatures and cannot account for the differences in the extent of differential expression between strains (Supplementary Table S2). Instead, these differences may be largely accounted for by higher inter-individual variation in gene expression in fish from the Coleman strain (Supplementary DataS2 at https://doi.org/10.5683/SP3/O0JVPC).

There were both shared and distinct responses to thermal acclimation between the strains (Fig. 4c). To examine the common core transcriptomic response to thermal acclimation, we performed gene ontology (GO) category enrichment on the set of DEGs shared in both strains (Fig. 4c; Supplementary DataS5 at https://doi.org/10.5683/SP3/O0JVPC). We did not detect significant enrichment for genes upregulated at 18°C compared to 12°C, and therefore, we examined the list of DEGs (Supplementary DataS4 at https://doi.org/10.5683/SP3/O0JVPC) for genes that we had *a priori* reason to believe might be involved in acclimation to these temperatures (Keen *et al.,* 2017). Consistent with the observed resetting of cardiac excitability by thermal acclimation, we detected multiple genes involved in cardiac excitation-contraction coupling such as the cardiac Ca^2+^ ATPase (ATP2A2 aka SERCA2), calsequestrin (CASQ2) and phospholamban (PLN), which are key regulators of cardiac intracellular Ca^2+^ levels. There were only a few enriched GO categories for genes downregulated at 18°C compared to 12°C (Supplementary DataS5 at https://doi.org/10.5683/SP3/O0JVPC), including categories associated with alternative splicing (GO:0000375), protein quality control (GO:0006515; GO:0070842) and epigenetic mechanisms such as gene silencing by RNA (GO:0031047).

Unlike the comparison between 12 and 18°C, there were many GO categories enriched in the DEGs between the acclimation temperatures of 18 and 24°C, and all were detected for the set of DEGs that were upregulated at 24°C (Fig. 4d). Many of these enriched GO categories were indicative of a cellular stress response at 24°C (Fig. 4d, Supplementary DataS5 at https://doi.org/10.5683/SP3/O0JVPC), with ~30% of the top 100 GO categories being related to protein folding and quality control, and containing many genes known to be associated with thermal stress, such as heat shock proteins. In addition, there was enrichment of a GO category associated with collagen biosynthesis (GO:0032964), which is known to be an important component of the response to temperature in the fish heart (Keen *et al.,* 2017; Johnston and Gillis, 2022).

Because the two strains differed significantly in gene expression at 18°C, we also performed GO enrichment analyses for both strains separately (Supplementary DataS6 at https://doi.org/10.5683/SP3/O0JVPC). In comparisons between fish acclimated to 12°C versus 18°C, there was limited GO enrichment in the Coleman strain. In the Shasta strain, however, there was significant GO enrichment for multiple categories among down regulated genes, including those involved in aerobic respiration (GO:0009060; GO0006120), along with a wide range of other mitochondrial processes, and those involved in protein folding and localization (Supplementary DataS6 at https://doi.org/10.5683/SP3/O0JVPC), suggesting that this strain may be making metabolic adjustments as a result of acclimation to low and moderate temperatures. Among genes upregulated between 12 and 18°C in the Shasta strain, there was enrichment for multiple GO terms associated with signal transduction, again suggesting a reorganization of cellular function. In comparisons between fish acclimated to 18°C versus 24°C, in the Coleman strain there was substantial enrichment for terms associated with mitochondrial metabolism and function in the down regulated genes, and there was evidence of enrichment for genes involved in cellular stress responses among the up-regulated genes (e.g. GO:0034976 response to endoplasmic reticulum stress; GO:0048102 autophagic cell death; GO:0034620; cellular response to unfolded protein). In the Shasta strain there was limited GO enrichment for down-regulated genes, but much greater enrichment among up-regulated genes, which was dominated by GO terms associated with a cellular stress response (e.g. GO:0061077 chaperone-mediated protein folding; GO:0061684 chaperone-mediated autophagy). Together, these data strongly indicate that both strains are experiencing cellular stress in the cardiac ventricle, at a high, but ecologically relevant acclimation temperature.

To further examine patterns of gene expression in response to thermal acclimation, we performed cluster analysis of patterns of differential expression independently in each strain (Supplementary Fig. S3). There were multiple clusters that exhibited substantial GO enrichment (Supplementary Data S7 at https://doi.org/10.5683/SP3/O0JVPC). For example, Coleman cluster 5, which represents genes that are progressively down-regulated with increasing temperature, included multiple GO terms related to central metabolic pathways, particularly those related to energy metabolism. Similarly, Coleman cluster 7, which represents genes that are down regulated at the lowest temperature also contained GO terms related to energy metabolism, and particularly those involved in mitochondrial electron transport and ATP synthesis. Together, these data point to profound reorganization of central metabolism with thermal acclimation in the Coleman strain. In the Shasta strain, on the other hand, there were clear indicators of a cellular stress response at high temperatures. For example, Cluster 1, which represents genes that are down-regulated at 24°C, was dominated by GO categories related to translation, suggestive of translational arrest at high temperature, and Cluster 7, which represents genes that are upregulated at 24°C, included GO categories related to endoplasmic reticulum stress and the heat shock response. In the Shasta strain, Cluster 3 also demonstrated significant GO enrichment. This cluster, which represents genes that increase in expression progressively with increasing temperature, was dominated by GO categories associated with histone and DNA methylation, suggesting an epigenetic component to the thermal acclimation response.

## Discussion

In the face of global climate change, an organism’s capacity for thermal acclimation may be a critical, but often neglected, predictor of species persistence (Somero, 2010; Gunderson *et al.,* 2017; Fox *et al.,* 2019; Morley *et al.,* 2019). For aquatic organisms, the capacity to simultaneously tolerate hypoxia may be of additional importance (Sampaio *et al.,* 2021). However, relatively few studies have attempted to examine the impacts of thermal acclimation on both thermal and hypoxia tolerance (Collins *et al.,* 2021), particularly close to the sustainable thermal limit. Here, we show that acclimation to increased temperature (from 12 to 18 to 24°C) improves both hypoxia and thermal tolerance in rainbow trout—combined compensatory responses that could partially buffer against the deleterious effects of climate change. However, compensation was incomplete for both traits, with a constraint on plasticity being particularly evident at the highest acclimation temperature we tested (24°C). Similarly, limits to AAS and anaerobic performance were also evident at this acclimation temperature. Moreover, we demonstrate similar limitations across levels of biological organization as indicators of cellular stress and oxygen limitation in the heart, limitations on the plasticity of cardiac function, and of whole-organism traits were all evident at the highest acclimation temperature. These results point to a pervasive thermal limit and limit to plasticity across multiple levels of biological organization at 24°C, a temperature that rainbow trout routinely experience during the summer months at their southern biogeographic range limit (Atkinson, 2018).

In addition, considering the data from fish acclimated to 18°C, there is clear alignment traits from metabolic rate, to thermal and hypoxia tolerance, to cardiac performance, to cardiac gene expression that shows differences between the two strains in their ability to cope with moderately high temperatures. However, at the highest acclimation temperature tested, both strains exhibit similar patterns of failure across levels of organization. As temperatures increase with ongoing climate change, we suggest that these strains of rainbow trout are unlikely to have sufficient plasticity to cope with temperatures that will become increasingly prevalent as climate change progresses (Dressler *et al.,* 2023).

### Thermal limits of aerobic and non-aerobic performance

Influential ideas such as the OCLTT hypothesis (Pörtner, 2002; Pörtner and Farrell, 2008) suggest that warming results in a decline in whole-animal physiological performance, as indicated by a progressive decline in AAS. AAS is an integrative trait that reflects the cardiorespiratory capacity to provide oxygen to support life-sustaining activities such as locomotion, growth and reproduction that are above maintenance requirements. Here we show that rainbow trout could not maintain AAS at the highest acclimation temperature tested. While the declines in AAS compared to peak levels were modest (18% in the Coleman strain and 29% in the Shasta strain), they still indicate that 24°C is a supra-optimal temperature, at which these fish cannot maintain peak aerobic scope, limiting maximum capacity to fuel activities aerobically, even in fully acclimated fish. Indeed, only slightly higher acclimation temperatures (25°C) can stop or reverse the growth of rainbow trout, including in the Shasta strain examined here (Myrick and Cech, 2000). Such comparisons not only emphasize that limitations on aerobic scope may be associated with reductions in other performance traits more closely related to fitness, but that the thermal-safety margin (TSM) for such traits may be very small. At high temperatures, fishes, including salmonids, are also known to increasingly rely on anaerobic metabolism to support performance (Brett, 1964; Jain and Farrell, 2003; Birnie-Gauvin *et al.,* 2023; Grimmelpont *et al.,* 2023). Similar to the loss in aerobic capacity at high temperatures, our data on excess post-exercise oxygen consumption (EPOC), an indirect indicator of anaerobic capacity, suggest that the capacity to support performance non-aerobically also reached its limit at our highest acclimation temperature pointing to critical failures in key pathways that support metabolism.

### Limits to plasticity in acute thermal and hypoxia tolerance

Heatwaves and hypoxic events have been suggested to be even more potent drivers of biodiversity change than gradual increases in mean temperature with climate change (Vasseur *et al.,* 2014; Román-Palacios and Wiens, 2020; Harvey *et al.,* 2022), emphasizing the potential importance of traits associated with short-term tolerance, such as the critical thermal maximum (CT_max_). Here we show that CT_max_ displayed modest plasticity in rainbow trout, as expected for a north-temperate zone fish (Morley *et al.,* 2019). However, its plasticity had clear limits as indicated by the substantial decline in the acclimation response ratio (ARR) between our highest two acclimation temperatures, such that the response is only about half that observed between the two lower acclimation temperatures. Similarly low ARR for CT_max_ with acclimation to high water temperature have been reported in multiple species of salmonids (Lee and Rinne, 1980). However, the ARR that we observe in warm-acclimated trout is in the lowest ~ 10% of all known ARRs for fishes (Comte and Olden, 2017b; Morley *et al.,* 2019). As a result, the TSM, or the difference between the maximum tolerated temperature (CT_max_) and the acclimation temperature, declined from over 16°C at the lowest acclimation temperature to less than 7°C at the highest acclimation temperature. Macro-physiological studies suggest that TSM may be a critical factor in determining vulnerability to climate change (Deutsch *et al.,* 2008; Huey *et al.,* 2009; Sunday *et al.,* 2014). Thus, the narrower TSM at the higher acclimation temperatures may place rainbow trout at risk at the southern end of the species range, given that these habitats already reach maximum temperatures from 31–33°C, and these streams show extensive diurnal variability that can exceed 6°C (Sloat *et al.,* 2013; Meza-Matty *et al.,* 2021; Dressler *et al.,* 2023). Thus, at the southern end of the species range trout may be exposed to temperatures that exceed the CT_Max_ of our warm-acclimated fish. Indeed, it has been shown that trout are unable to persist across years in streams that reach these temperatures (Sloat *et al.,* 2013).

In natural environments, high temperature events are often accompanied by episodes of low oxygen. Consequently, the ability to induce the type of plasticity known as cross-tolerance and thus increase hypoxia tolerance when acclimated to warm temperatures is likely to be highly beneficial for aquatic organisms. However, there is substantial uncertainty as to whether warm-acclimation improves hypoxia tolerance in fishes (Collins *et al.,* 2021; Earhart *et al.,* 2022), largely because very few studies have used experimental designs adequate to disentangle the acute effects of high temperature from the effects of acclimation on hypoxia tolerance. Here, we characterized the effects of thermal acclimation on hypoxia tolerance using two complementary metrics: LOE_hyp_ and P_crit_. While LOE_hyp_ depends on both oxygen supply and anaerobic capacity, P_crit_ is shaped by oxygen supply capacity under a less severe hypoxic condition (Nilsson and Östlund-Nilsson, 2008; Mandic *et al.,* 2009; Ultsch and Regan, 2019). Our study indicates that these two hypoxia-tolerance traits differ in their acclimation capacity. Acclimation to 18°C resulted in complete (Shasta strain) or nearly complete (Coleman strain) compensation of LOE_hyp_, whereas P_crit_ demonstrated only partial compensation of a similar extent in both strains. However, it is important to note that we only measured acclimation capacity directly for LOE_hyp,_ whereas for P_crit_ we inferred constrained acclimation capacity based on the inability to maintain P_crit_ in fully acclimated fish at the three acclimation temperatures. Despite this limitation, our data suggest that rainbow trout have greater capacity to adjust oxygen-independent metabolism than oxygen uptake during acclimation to intermediate temperatures. Yet, after acclimation to 24°C, a clear limit to plasticity in hypoxia tolerance again emerged; both LOE_hyp_ and P_crit_ increased substantially at our highest acclimation temperature, indicating that plasticity is limited at this high but ecologically relevant temperature. This limit to the plasticity of hypoxia tolerance is especially important because meta-analyses of the impacts of global change suggest that realistic changes in oxygen availability often have larger effects on growth, development, survival and reproduction than do realistic changes in temperature for aquatic organisms such as fish (Sampaio *et al.,* 2021).

### Changes in cardiac function and gene expressions support organismal thermal limits

The maintenance of cardiac function with warming is essential to support adequate oxygen and metabolite transport around the body (Farrell *et al.,* 2009). In fishes, the elevated aerobic metabolic demand with warming is generally met through increasing cardiac output via increased heart rate (Steinhausen *et al.,* 2008). However, maximum heart rate (ƒ_Hmax_) can only increase up to a point with acute warming (peak ƒ_Hmax_ at T_peak_), beyond which it plateaus or declines, and the heartbeat eventually becomes arrhythmic (T_arr_). This impaired function then constrains cardiac output and aerobic capacity (Farrell, 2016). As is the case for many fish species (Casselman *et al.,* 2012; Anttila *et al.,* 2014; Gilbert and Farrell, 2021; Adams *et al.,* 2022), rainbow trout exhibited substantial plasticity in cardiac thermal tolerance with both T_peak_ and T_arr_ increasing with acclimation temperature. This plasticity is likely essential given that we observed impairments in cardiac function during acute warming below our upper acclimation temperature in trout acclimated to 12°C. Such a cardiac limitation would constrain convective oxygen transport and thus could help explain why whole-animal hypoxia tolerance was so poor when 12°C-acclimated fish were acutely tested at 24°C as compared with those acclimated to 24°C, and why hypoxia tolerance improved with warm acclimation. However, in rainbow trout, as in other species, the compensation of cardiac heat tolerance was incomplete. Consequently, the TSM for T_arr_ decreased as acclimation temperature increased. For example, these two rainbow trout strains acclimated to 24°C would experience loss of cardiac function with only 4–6°C of acute warming, which is already within the range of diel temperature fluctuations experienced by some populations (Rodnick *et al.,* 2004; Dressler *et al.,* 2023).

Consistent with observations in other fish species (Gilbert and Farrell, 2021), warm-acclimation of rainbow trout also resulted in a substantial resetting of ƒ_H_ to lower levels over intermediate test temperatures. This compensatory resetting of ƒ_Hmax_ observed for warm-acclimated rainbow trout appears to be a consistent response to warm acclimation across salmonid species (Anttila *et al.,* 2014; Drost *et al.,* 2016; Gilbert and Farrell, 2021), including in two other strains of rainbow trout (Adams *et al.,* 2022; Gilbert *et al.,* 2022). In all of these cases, ƒ_Hmax_ was reset during warm acclimation to a similar extent to that seen here, when compared over similar changes in temperature. Our data are also consistent with a general pattern that the peak ƒ_Hmax_ achieved during acute warming can increase following warm acclimation, these two factors resulted in warm acclimation improving Δƒ_Hmax_. Together, the observed plasticity in ƒ_Hmax_ with warm acclimation likely allows fish to better balance increases in cardiac stroke volume and heart rate during routine locomotor activity at a given acclimation temperature, while the improvement or maintenance of Δƒ_Hmax_ could help trout cope with acute heat exposure. Nonetheless, with most cardiac metrics (e.g. absolute Δƒ_Hmax_, peak ƒ_Hmax_ and T_arr_ in Shasta trout; Fig. 3) reaching a ceiling for thermal acclimation, the limits on cardiac thermal plasticity align with those we observed for whole-animal hypoxia and heat tolerance.

Our results at the whole-animal and organ level provide strong support for a limit on plasticity that may be important in determining the performance of rainbow trout in a changing environment. At the cellular and molecular level, our transcriptomic results lend considerable support to this thesis. The changes in cardiac transcript levels at moderate acclimation temperatures suggest that the heart undergoes significant metabolic remodelling and makes adjustments in the expression of genes involved in excitation–contraction coupling, which would be consistent with a beneficial acclimation response. By contrast, at the highest acclimation temperature, clear evidence of a significant cellular stress response was apparent with upregulation of genes associated with endoplasmic reticulum stress and the heat shock response. Although RNA expression levels are not always predictive of changes in protein amount or activity (Schwanhäusser *et al.,* 2011; Buccitelli and Selbach, 2020), correlations between these steps in the gene expression cascade are reasonably high and are typically strongest under steady-state conditions (Liu *et al.,* 2016), such as following thermal acclimation. In addition, although it is difficult to predict the extent of correlation between mRNA, protein and biochemical activity for any particular gene, when multiple genes involved in a biological process exhibit differences in mRNA levels as observed here, it is even more likely that the function of this process is being affected by acclimation. Indeed, similar transcriptomic patterns with exposure to elevated temperatures at both acute and chronic time scales have been observed (Narum and Campbell, 2015; Komoroske *et al.,* 2021; Durhack *et al.,* 2025). However, it should be noted that interpreting the functional significance of the appearance of a cellular stress response is challenging, as these changes could be indicators of cellular damage, or could represent a beneficial repair response. These results highlight both the utility and complexity of transcriptomic approaches in conservation contexts (Connon *et al.,* 2018).

### Relevance to other strains of rainbow trout

Determining whether other strains of rainbow trout may differ in their thermal plasticity compared to the strains examined here is critically important for forecasting the vulnerability of the species as a whole (McKenzie *et al.,* 2020; Schulte and Healy, 2022). We contend that our data on the limits of plasticity in these strains may represent a best-case scenario for this species because comparison with data from other trout strains suggest that the strains we examined are warm-adapted, especially the Coleman strain, which also had a greater hypoxia tolerance. For example, Redband trout, a rainbow trout subspecies that has adapted to the hot, arid, conditions of eastern Oregon (Narum *et al.,* 2010), have greater thermal tolerance than other populations in Oregon (Rodnick *et al.,* 2004; Chen *et al.,* 2015), but their CT_max_ is very similar to that of our California strains. Similarly, wild rainbow trout from the extreme southern end of their distribution in California also have a CT_max_ that is very similar to those reported here for warm-acclimated trout (Dressler *et al.,* 2023). This suggests that there may be limited opportunity to increase CT_max_ in rainbow trout through evolutionary adaptation beyond the levels observed here. Furthermore, while both intentional and unintentional selection at high temperatures of rainbow trout in aquaculture and hatchery settings has led to increases in thermal tolerance, the selected lines have a similar CT_max_ to that of the strains examined here. For example, a rainbow trout line that has been under selection in Japan since 1966 has a CT_max_ of 30.0°C when acclimated to 20°C (Ineno *et al.,* 2018). This CT_max_ is similar to that predicted for our two strains at a 20°C acclimation temperature (i.e. 30.1 and 30.5°C for Shasta and Coleman strains, respectively). Thermally selected rainbow trout strains in Patagonia have a CT_max_ between 29 and 29.3°C when acclimated to ~ 20°C (Crichigno *et al.,* 2018), which is lower than the CT_max_ for our California strains. A particularly interesting comparison can be made with hatchery rainbow trout in Western Australia that have undergone intense selection on thermal tolerance due to natural high temperature events (Adams *et al.,* 2022). This strain has a California origin and, despite strong thermal selection, CT_max_ and the upper thermal limits for cardiac function (T_arr_, T_peak_) are remarkably similar to those measured here (Adams *et al.,* 2022). Taken together, these data suggest that opportunities for rainbow trout to increase upper thermal tolerance (as measured by CT_max_ and cardiac performance) beyond that measured in our California hatchery strains are likely limited.

In addition to genetic variation among strains, various types of plasticity acting at different timescales have the potential to alter thermal tolerance or performance or their plasticity (Schulte and Healy, 2022). For example, our study did not assess whether either early developmental or transgenerational plasticity could provide an additional buffer against climate change beyond the plasticity we observed. All the same, while the developmental environment has been shown to alter thermal tolerance in zebrafish (Schaefer and Ryan, 2006), the extent of this plasticity is very small compared to the effects of acclimation. Indeed, meta-analyses suggest that the extent of developmental plasticity in response to temperature is limited and does not persist across the lifespan in most ectotherms (Vagner *et al.,* 2019; Pottier *et al.,* 2022). Consequently, developmental plasticity both for thermal and hypoxia tolerance is generally thought to provide very little beneficial effect (Earhart *et al.,* 2022). Less is known about the potential for transgenerational plasticity to improve performance in a warming world, although most studies suggest that the capacity for this type of plasticity is also limited (Uller *et al.,* 2013; Donelson *et al.,* 2018).

## Conclusions and Implications for Conservation

As we proceed through this time of climatic warming, our results suggest that the phenotypic plasticity of rainbow trout in response to increasing temperature will be insufficient to maintain adequate performance of these fish in their natural habitat at the southern end of their range as climate warms, which suggests that the range limit will move northward. This will limit the waterbodies that can sustain rainbow trout for management and recreational purposes, as well as constraining their natural distributions. Notably, we focused on rainbow trout strains that are relatively warm-adapted and strains that are less warm-adapted (such as those in the Pacific Northwest; (Strowbridge *et al.,* 2021)) are likely to be even more constrained.

Our work demonstrates the importance of examining multiple traits and integrating measurements of plasticity across levels of biological organization for predicting species response to climate change. Understanding thermal limits and the limits to plasticity is critically important for managers of freshwater fisheries, as well as for those who control water use for agriculture and human consumption in drought conditions, as water-use management and fisheries conservation increasingly come into conflict.

## Supplementary Material

Web_Material_coaf034

## Data Availability

All Supplementary Figures, Tables, raw data and code are available in the Canadian Data Repository, Borealis at https://doi.org/10.5683/SP3/O0JVPC
