# Supplementary material for: Climate warming will test the limits of thermal plasticity in rainbow trout, a globally distributed fish
Source: Conserv Physiol. 2025 May 28;13(1):coaf034. doi: 10.1093/conphys/coaf034 (PMC12257943; doi:10.1093/conphys/coaf034)
Supplement: Web_Material_coaf034 [file web_material_coaf034.pdf]

Supplemental Figures and Tables for: Climate warming will test the limits of thermal plasticity in rainbow trout, a globally distributed fish

N. Strowbridge, M.J.H. Gilbert, Y. Zhang, D.C.H. Metzger, J.L. McKenzie, L. Lima, A.P. Farrell, N.A. Fangue & P.M. Schulte

This file contains Supplemental Figures 1-3 and Supplemental Tables 1 & 2

For all raw data and code please see:

https://borealisdata.ca/dataset.xhtml?persistentId=doi:10.5683/SP3/O0JVPC

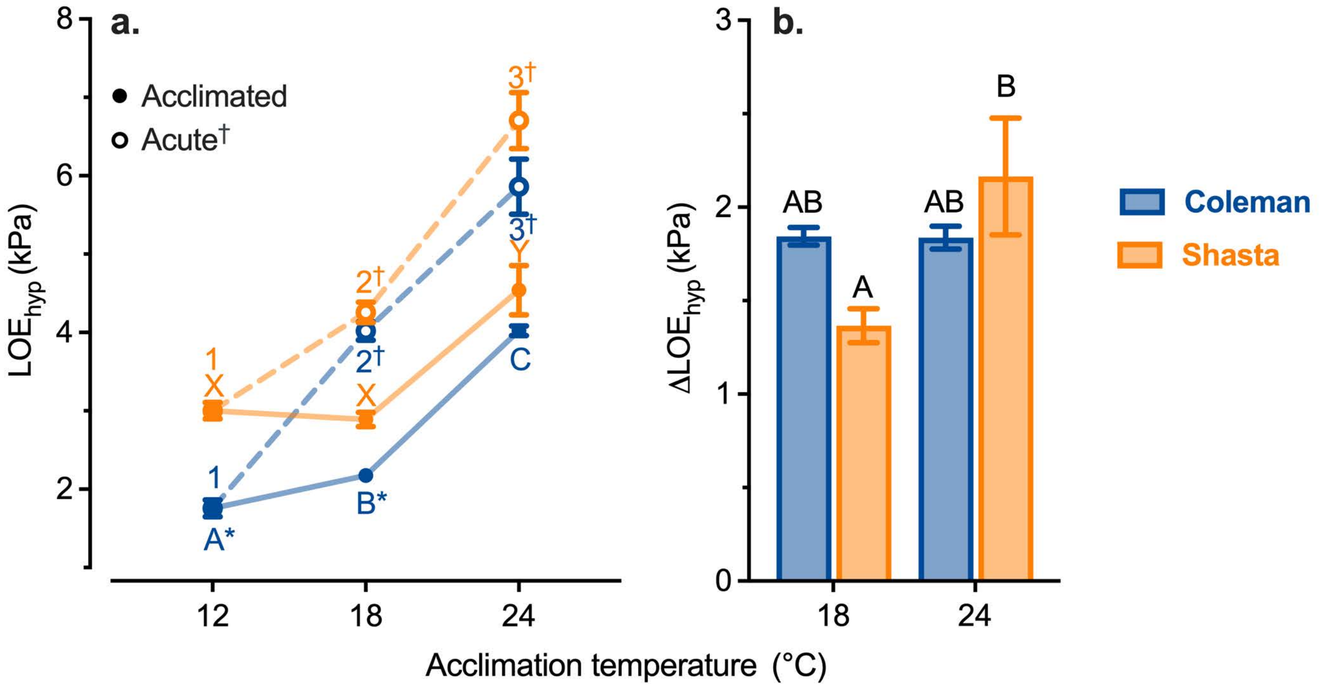

Fig S1. Effects of thermal acclimation on the hypoxia tolerance of two strains of rainbow trout. Panel a. Hypoxia tolerance of (Coleman (blue) and Shasta (orange)) trout strains measured as the oxygen tension at which fish exhibit loss of equilibrium (LOE_{hyp}). Note that low LOE_{hyp} reflects high hypoxia tolerance. Closed symbols reflect the LOE_{hyp} of fish tested at their acclimation temperature. Open symbols reflect the LOE_{hyp} of fish acclimated to 12 ºC that were acutely transferred to either 18 or 24 ºC and tested at that temperature. Dissimilar letters indicate significant differences within a strain between acclimation temperatures (Coleman: A,B,C; Shasta: X,Y,Z). Dissimilar numbers represent significant different within a strain for acutely transferred fish. Panel b. The change in LOE_{hyp} between acutely transferred and acclimated fish (LOE_{hyp} acute- LOE_{hyp} acclimated), which is an index of the effect of acclimation. Dissimilar letters indicate significant differences between groups. All data are mean ± SEM

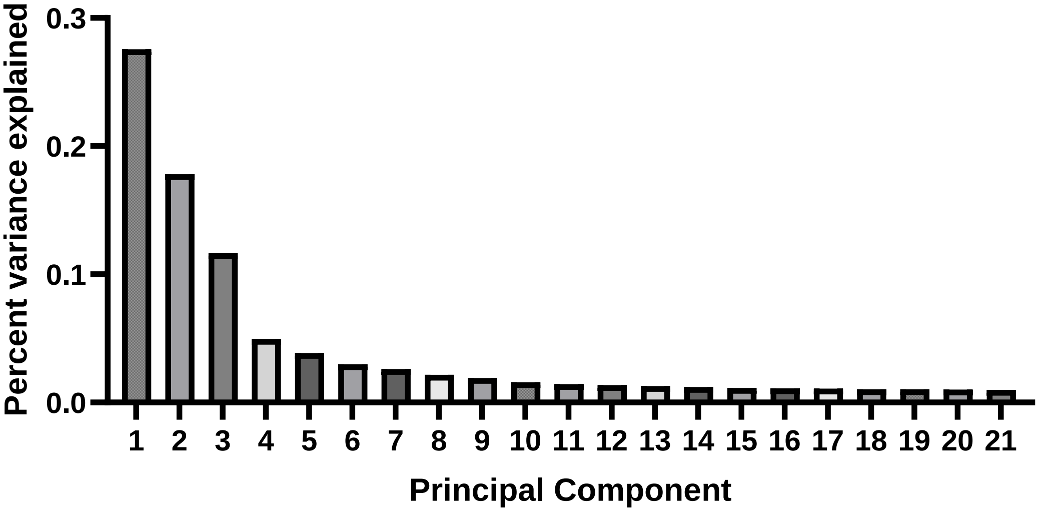

Figure S2: Percent variance explained for all Principal Components for gene expression in cardiac ventricle of two California rainbow trout strains (Shasta and Coleman) acclimated to 12, 18 and 24 °C.

Figure S3: Cluster analysis of cardiac gene expression in rainbow trout acclimated to 12, 18 and 24 ℃

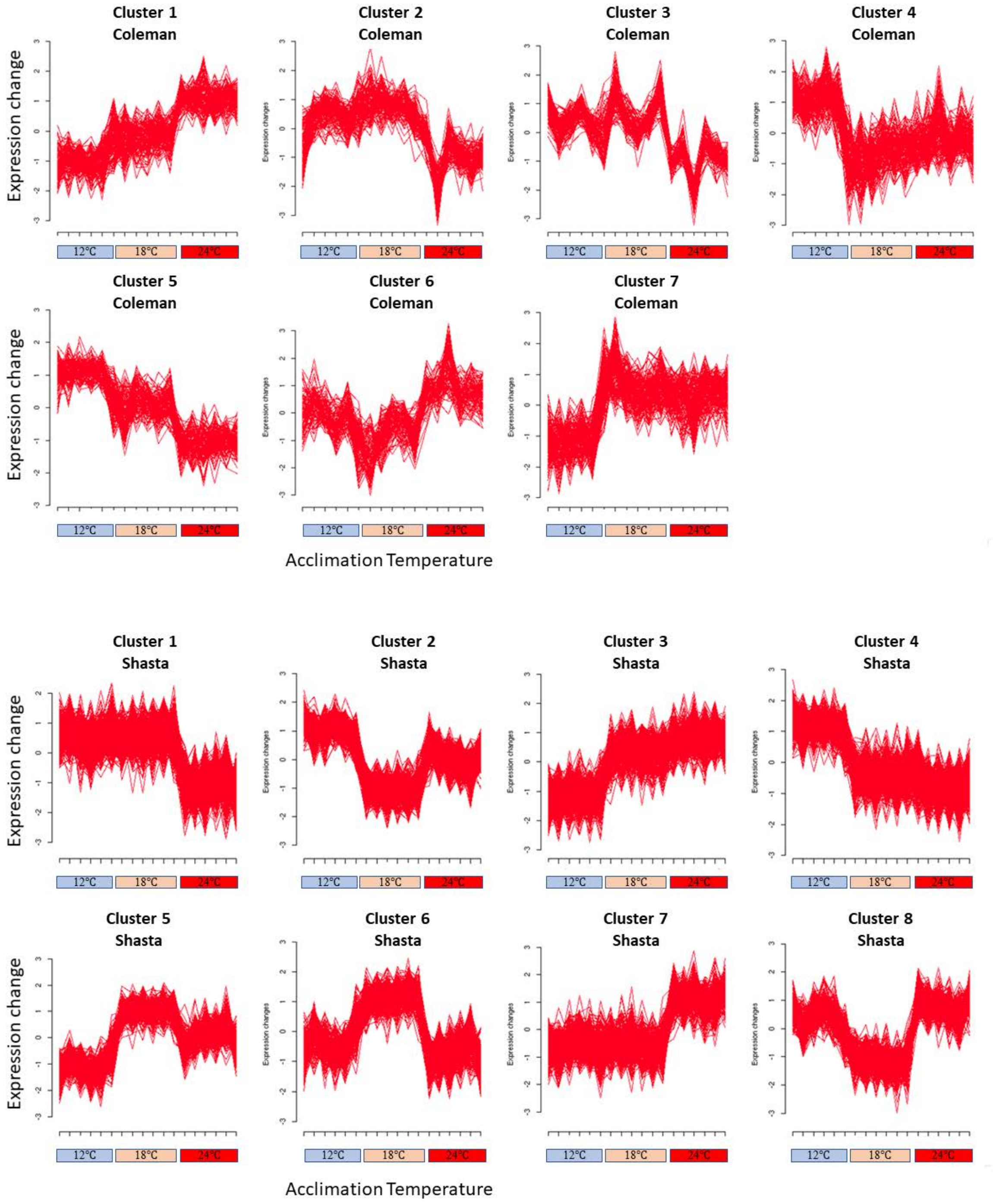

Supplemental Table 1. Sample size (n), mass and length for fish used in each experiment within the study. Data are shown as mean ± s.e.m.

		Shasta			Coleman		
	Temperature (°C)	n	Mass (g)	Length (mm)	n	Mass (g)	Length (mm)
CT_{max}	12	12	43.2 ± 4.8	156.4 ± 6.3	12	4.3 ± 0.2	73.2 ± 1.5
	18	12	56.4 ± 4.2	177.8 ± 2.8	12	4.7 ± 0.4	74.7 ± 2.3
	24	12	51.9 ± 4.3	167.1 ± 5.0	12	4.5 ± 0.4	70.2 ± 1.5
ILOS	12	12	53.3 ± 5.2	170.0 ± 6.1	12	4.1 ± 0.2	70.9 ± 1.4
	18	12	55.9 ± 4.5	174.7 ± 4.9	12	6.3 ± 0.6	80.8 ± 2.1
	24	12	49.5 ± 4.2	164.3 ± 5.3	12	3.6 ± 0.4	65.6 ± 2.2
Acute ILOS	12-18	12	50.3 ± 4.8	167.6 ± 5.9	12	4.8 ± 0.3	77.0 ± 1.5
	12-24	12	63.4 ± 6.1	182.1 ± 5.9	12	5.4 ± 0.4	76.6 ± 2.2
IRAP	12	13	41.4 ± 2.0	-	13	4.2 ± 0.3	-
	18	14	49.2 ±2 .0	-	14	4.8 ± 0.3	-
	24	14	48.5 ± 2.0	-	12	4.3 ± 0.3	-
Cardiac thermal tolerance	12	8	39.8 ± 3.5	153.9 ± 5.6	11	4.6 ± 0.4	71.4 ± 2.1
	18	8	46.4 ± 5.7	163.9 ± 7.1	10	5.5 ± 0.6	73.8 ± 2.8
	24	8	47.2 ± 4.8	164.5 ± 5.6	8	4.6 ± 0.5	70.3 ± 2.4
Cardiac RNAseq	12	6	57.9 ± 5.9	173.8 ± 5.7	6	4.9 ± 0.7	72.2 ± 3.2
	18	6	51.7 ± 8.3	169.7 ± 8.3	6	7.8 ± 1.1	82 ± 4.0
	24	6	42.2 ± 2.8	156.5 ± 4.6	6	5.0 ± 0.3	71 ± 1.6

CT_{max} : Critical thermal maximum
ILOS : Incipient lethal oxygen saturation
IRAP: Integrated respiratory assessment paradigm

Supplemental Table 2: RNA-sequencing read depth for all samples

Sample[1]	Raw Reads #	Surviving Reads #	Surviving %
12C_CH1	17537475	16281262	92.84
12C_CH3	16485374	15172353	92.04
12C_CH5	21181806	19710099	93.05
12C_CH6	19221831	17953184	93.4
12C_CH7	13863376	12891907	92.99
12C_CH8	17384392	16138079	92.83
12C_SH1	20856095	19457170	93.29
12C_SH2	18711718	17526472	93.67
12C_SH3	16969735	15992621	94.24
12C_SH4	20730156	19563816	94.37
12C_SH5	19217809	18207793	94.74
12C_SH6	19791921	18572510	93.84
18C_CH1	6568483	3205586	48.8
18C_CH3	15692729	14602979	93.06
18C_CH4	21076917	19779304	93.84
18C_CH6	16734028	15457619	92.37
18C_CH7	15498696	14415917	93.01
18C_CH8	17632843	16400618	93.01
18C_SH1	22398846	20979841	93.66
18C_SH2	20660658	19311505	93.47
18C_SH3	13977429	13040227	93.29
18C_SH4	19511141	18304831	93.82
18C_SH7	20144326	19009758	94.37
18C_SH8	18722298	17637862	94.21
24C_CH1	17548124	16353504	93.19
24C_CH3	17118662	15920355	93
24C_CH4	16686834	15658522	93.84
24C_CH5	17457502	16246550	93.06
24C_CH6	17925186	16088797	89.76
24C_CH8	19526333	18105118	92.72
24C_SH1	21296540	19933645	93.6
24C_SH2	21832996	20623139	94.46
24C_SH3	19032455	17964728	94.39
24C_SH4	16495024	15297959	92.74
24C_SH5	21181806	19710099	93.05
24C_SH7	22128095	20725236	93.66

1: Sample naming convention: Acclimation temperature (°C) population (CH= Coleman, SH=Shasta)_individual ID number
